# Inflammaging: triggers, molecular mechanisms, immunological consequences, sex differences, and cutaneous manifestations

**DOI:** 10.3389/fimmu.2025.1704203

**Published:** 2025-12-05

**Authors:** Ebru Karpuzoglu, Steven D. Holladay, Robert M. Gogal

**Affiliations:** Department of Biomedical Sciences, College of Veterinary Medicine, University of Georgia, Athens, GA, United States

**Keywords:** inflammaging, immunosenescence, sex differences, estrogen, skin aging, inflammation

## Abstract

Inflammaging, defined as chronic, low-grade, systemic inflammation that increases with age in the absence of overt infection, is a phenomenon that was first described in 2000 as a member of a growing number of age-related processes that had pleiotropic effects on immune function and disease susceptibility. Although many pathological consequences have been attributed to inflammaging, it remains distinct from immunosenescence and not completely understood. A resurgence of interest in inflammaging has been spurred by recent work demonstrating roles for senescent cells in driving chronic inflammatory signaling and defining the cellular and molecular triggers that sustain cytokine production during aging. Alongside elevations in pro-inflammatory mediators (e.g., IL-6, TNF-α, IL-1β), attention to anti-inflammatory mediators (e.g., IL-10, IL-1Ra) and composite ratios (e.g., IL-6:IL-10) can better index inflammatory balance in older adults. In this review, we summarize the characterization of inflammaging mechanisms, highlight roles for chronic inflammation that are clearly defined in immune system remodeling, and outline questions regarding inflammaging functions in sex differences, hormonal regulation, autoimmunity, and skin biology that still require further exploration.

## 1 Inflammaging vs. immunosenescence

Inflammaging, first defined in 2000 by Franceschi and colleagues, refers to chronic, low-grade, systemic inflammation that increases with age, even in the absence of overt infection or disease (1). It is characterized by elevated pro-inflammatory cytokines (e.g., IL-6, TNF-α, IL-1β), chemokines, acute-phase proteins, and accumulation of senescent cells with a pro-inflammatory senescence-associated secretory phenotype known as SASP (2–5). Inflammaging is recognized as a force driving age-related diseases, including cardiovascular, neurodegenerative, metabolic, and autoimmune disorders (1, 4).

Immunosenescence describes a functional decline of the immune system affecting both innate and adaptive arms (5–7). Hallmarks include thymic involution, reduced naïve T and B cell output, memory and senescent lymphocyte expansion, impaired antigen presentation, and dysregulated cytokine production (4, 6, 7). Immunosenescence increases susceptibility to infections and reduces vaccine responsiveness, while inflammaging contributes to tissue damage and age-related pathology (3, 5–7). Although immunosenescence and inflammaging are distinct phenomena, they are mechanistically interconnected as senescent immune cells promote inflammaging through the SASP, while chronic inflammation accelerates immune exhaustion and dysfunction (3, 5–7).

## 2 Triggers of inflammaging

Inflammaging arises from intrinsic aging processes and extrinsic environmental factors converging on molecular pathways and understanding these triggers is essential for identifying intervention points in age-related diseases.

### 2.1 Internal (aging-related) factors

Senescent cells accumulate with age due to repeated cellular stress, telomere attrition, DNA damage, and oncogenic signaling that can trigger inflammaging, These cells adopt a distinct phenotype with permanent cell cycle arrest and SASP with pro-inflammatory cytokines (IL-6, IL-1β, TNF-α), chemokines, growth factors, proteases driving local and systemic inflammation Coppé et al. showed that oncogene-induced senescent cells secrete IL-6 and IL-8, while Acosta et al. demonstrated that IL-1 signaling reinforces the senescent growth arrest (8, 9). SASP reinforces senescence in an autocrine manner and induces paracrine-driven inflammation to neighboring cells and throughout the organism, contributing to tissue dysfunction, immune evasion, and tumor promotion.

Key SASP molecular regulators include NF-κB, C/EBPβ, and mTOR signaling, which integrate stress signals and drive inflammatory mediator transcription. SASP is modulated by persistent DNA damage responses, dysfunctional mitochondria, and altered chromatin structure, including Lamin B1 loss and senescence-associated heterochromatin foci formation (10–12).

SASP-secreting senescent cells accumulation is implicated in pathogenesis of multiple age-related diseases, including osteoarthritis, atherosclerosis, neurodegeneration, and cancer and are under investigation to improve health span.

#### 2.1.1 Mitochondrial dysfunction and DAMP generation

Mitochondria are central hubs of cellular metabolism and innate immune signaling. With age, mitochondrial dysfunction becomes pervasive involving impaired oxidative phosphorylation, increased reactive oxygen species (ROS) production, and mitochondrial damage-associated molecular patterns (mtDAMPs) release, like mitochondrial DNA (mtDNA) and cardiolipin (13, 14). Damaged mitochondria release mtDNA and ROS acting as DAMPs activating innate immune sensors like NLRP3 inflammasome (12, 15, 16).

Mitochondrial ROS and mtDAMPs-activated NLRP3 inflammasome is a multiprotein complex that catalyzes maturation of pro-inflammatory cytokines IL-1β and IL-18 via caspase-1 activation (17, 18). Impaired mitophagy amplifies this process leading to dysfunctional mitochondria accumulation and a feedforward inflammation loop. Mitochondrial dysfunction intersects with the cGAS-STING and NF-κB pathways amplifying inflammatory responses, where cGAS-STING detects cytoplasmic DNA and activated nuclear NF-κB stimulates proinflammatory cytokine production, contributing to cardiovascular, neurodegenerative, metabolic, and malignant diseases and making mitochondrial health promising targets for inflammaging intervention (18).

#### 2.1.2 Accumulation of damaged macromolecules and other self-debris particles

Aging is associated with progressive accumulation of damaged proteins, lipids, nucleic acids, and cellular debris with declining proteostasis and autophagy. Inefficient apoptotic cell and cellular debris clearance fuel chronic innate immunity activation. Hanayama et al. demonstrated that defective clearance of apoptotic cells leads to chronic inflammation in mouse models (19). These endogenous molecules act as DAMPs activating pattern recognition receptors (PRRs) such as Toll-like receptors (TLRs) and NOD-like receptors (NLRs) on innate immune cells perpetuating inflammaging. Zhang et al. showed that mitochondrial DAMPs activate neutrophils through TLR9, while Iyer et al. demonstrated that necrotic cells release alarmins that trigger inflammation (14, 20). Persistent PRRs activation by self-DAMPs can result in chronic NF-κB signaling, increased cytokine production, and impaired inflammation resolution.

#### 2.1.3 Gut microbiota dysbiosis and increased intestinal permeability

Gut microbiota undergoes compositional changes with age often shifting toward a pro-inflammatory profile and losing beneficial commensals (21, 22). These age-related changes can increase gut permeability and systemic exposure to microbial PAMPs perpetuating inflammation (23–25). This dysbiosis impairs intestinal barrier integrity, increasing permeability (“leaky gut”) and allowing microbial product translocation such as lipopolysaccharides (LPS) into the circulation (24, 25). LPS and other microbial products activate TLRs, upregulating systemic cytokines (e.g., TNF-α, IL-6, IL-1β) and promoting a chronic pro-inflammatory state characteristic of inflammaging (24–26). Dysbiosis also affects immune cell function, reducing myeloid cells’ ability to clear senescent or apoptotic cells, further exacerbating inflammation. Therapeutic interventions targeting the microbiome (e.g., prebiotics, probiotics, fecal microbiota transplantation) are currently being explored to counteract this inflammatory profile.

#### 2.1.4 Oral microbiota and inflammaging

The oral microbiota have been shown to undergo changes with aging that could contribute to inflammaging. Age-related shifts in oral microbial composition, including increased prevalence of periodontal pathogens such as Porphyromonas gingivalis have been observed in older adults (65–80 years) correlating with elevated serum IL-6 and CRP compared to younger adults (27). Experimental gingivitis in healthy older adults induces systemic endotoxemia with plasma endotoxin increasing from 50 to 201 pg/mL and corresponding rises in soluble CD14 (28). Treatment of periodontitis reduces inflammatory markers in serum CRP and IL-6 after 2 months (29).

#### 2.1.5 Complement and coagulation system chronic activation

Complement system is a major contributor to both inflammation and thrombosis. With age, complement activation dysregulation leads to increased generation of small protein fragment anaphylatoxins, primarily C3a, C4a, and C5a, which bind to their receptors on immune and endothelial cells, promoting cytokine release, leukocyte recruitment, and increased endothelial permeability (30). In healthy individuals aged 70–85 years, plasma C3a levels were significantly elevated (mean 142 ng/mL) compared to young adults aged 20-35 (mean 67 ng/mL), and C3a levels correlated with IL-6 and TNF-α (31).

Terminal complement complexes (C5b-9) can also activate platelets and endothelial cells, fostering a prothrombotic state. Increased coagulation activity and defective complement regulation then amplify these inflammatory cascades (30, 32).

Similarly, coagulation pathways are linked to inflammation. Inflammaging is associated with increased coagulation activity and impaired fibrinolysis, which, in turn, amplify inflammatory signaling through protease-activated receptors (PARs) and tissue factor expression (33). Complement and coagulation dysregulation may then contribute to the pathogenesis of age-related vascular diseases, including atherosclerosis, thrombosis, and heart failure.

### 2.2 Environmental and lifestyle factors in inflammaging

Environmental and lifestyle factors such as diet, pollution, stress, and chronic infections play a pivotal role in inflammaging by persistently activating immune and inflammatory pathways.

#### 2.2.1 Diet and glycation end products

Dietary patterns rich in saturated fats and sugars accelerate inflammaging by promoting advanced glycation end-products (AGEs) formation. AGEs bind to the receptor for AGEs (RAGE) on immune and endothelial cells, triggering NF-κB-mediated pro-inflammatory cytokine transcription (34). Studies demonstrate that high-AGE diets rich in high-fat, high-sugar increase circulating TNF-α, C-reactive protein, and vascular adhesion molecules, while AGE restriction reduces these markers (24, 34). Visceral adiposity is a potent source of pro-inflammatory cytokines and chemokines, with increased infiltration of immune cells and senescent macrophages in adipose tissue (35, 36). Therefore, dietary modification to reduce AGE intake and the volume of adiposity may help systemic inflammaging attenuation lowering the risk of age-related diseases.

#### 2.2.2 Obesity and sedentarism

Obesity represents a major driver of inflammaging through adipose tissue dysfunction. And visceral adiposity that can secrete pro-inflammatory adipokines including IL-6, TNF-α, and leptin, while reducing anti-inflammatory adiponectin (37, 38). Obese individuals showed increased macrophage infiltration in adipose tissue, with a shift from anti-inflammatory M2 to pro-inflammatory M1 phenotype (39). Sedentary lifestyle was associated with higher levels of leptin and TNF-α, and lower adiponectin-to-leptin ratios, independent of moderate-to-vigorous physical activity levels, suggesting a direct link between sedentary behavior and a pro-inflammatory profile (40).

#### 2.2.3 Environmental pollution

Environmental pollutant exposure has been shown to increase oxidative stress and systemic inflammation due to industrialized lifestyles and varies significantly across global populations (41). Air pollutants including particulate matter (PM), nitrogen oxides, and volatile organic compounds may contribute to oxidative stress induction and mitochondrial dysfunction via activating NF-κB and MAPK pathways in immune and epithelial cells (42, 43). Pollutants can activate aryl hydrocarbon receptor (AhR) promoting Th17-mediated inflammation and suppressing regulatory T cell (Treg) function exacerbating autoimmunity and chronic inflammation (43, 44). Pollutants can also induce epigenetic modifications, including altered DNA methylation and histone modifications, which further dysregulate immune responses and contribute to premature aging (43, 45). Chronic exposure to pollution is associated with a heightened incidence of cardiovascular, respiratory, metabolic, and autoimmune diseases in the older adults, positioning this factor as an environmental inflammaging driver.

#### 2.2.4 Psychological stress

Chronic psychological stress has been shown to be a potent activator of the inflammasome and pro-inflammatory cytokine cascades that elevates systemic cytokine levels. Iwata et al. demonstrated that psychological stress activates the NLRP3 inflammasome through ATP release and P2X7 receptor activation, leading to IL-1β production (46). Frank et al. showed that stress-induced glucocorticoid resistance results in sustained elevation of IL-6 and TNF-α (47). These stress-induced mechanisms may collectively accelerate inflammaging in young adults and are linked to a higher risk of depression, cardiovascular disease, and metabolic syndrome in older adults. **Table 1** provides an overview of key triggers and pathways in inflammaging, including internal factors and external factors.

**Table 1:** Key triggers and pathways in inflammaging.

Trigger	Molecular pathways involved	Clinical Implications	References
Cellular senescence/SASP	NF-κB, mTOR, DNA damage	Tissue dysfunction, cancer, frailty	(26, 162)
Mitochondrial dysfunction	ROS, NLRP3, cGAS-STING	CVD, neurodegeneration, metabolic syndrome	(2, 26, 162)
Damaged macromolecules/DAMPs	PRRs (TLRs, NLRs), NF-κB	Atherosclerosis, Alzheimer’s	(2, 26, 162)
Gut microbiota dysbiosis	TLRs signalling	Metabolic, autoimmune diseases	(24, 163–165)
Complement/coagulation dysregulation	C3a/C5a, PARs	Thrombosis, atherosclerosis	(2, 26)
AGEs (Diet)	RAGE, NF-κB	Systemic inflammation, vascular disease	(2, 24, 26)
Environmental pollution	NF-κB, MAPK, AhR	Respiratory, metabolic disease	(2, 26)
Psychological stress	NLRP3, IL-1β	Depression, metabolic syndrome	(2, 26)
Chronic infections	Persistent antigenic stimulation, cytokines	Immunosenescence, increased disease risk	(2, 26)

## 3 Impact of inflammaging on innate immunity

Aging profoundly alters innate immunity function, phenotype, and molecular signaling of key cellular players. Inflammaging is both a driver and a consequence of these changes with homeostatic shift in innate responses and thus, the predisposition of older individuals to infection, chronic disease, and impaired tissue repair (4, 7, 48).

### 3.1 Macrophages: central effectors and molecular pathways

Macrophages are central to innate immune response orchestration and are critically involved in inflammation initiation and resolution. In inflammaging, macrophages undergo functional reprogramming, characterized by a low-level activation state and an altered polarization profile (4, 49–51). Aging impairs macrophage polarization, often skewing toward a pro-inflammatory phenotype with increased basal secretion of IL-1β, TNF-α, IL-6, CXCL9 and CXCL10, even in the absence of overt stimulation (52, 53). Zhang et al. demonstrated increased basal IL-1β and TNF-α secretion in aged macrophages, while Becker et al. showed altered polarization profiles (52, 53). This is driven in part by mitochondrial dysfunction, notably reduced mitochondrial calcium (mCa^2+^) uptake, which amplifies cytosolic calcium signaling and activates the NF-κB pathway, rendering macrophages hyper-responsive to inflammatory stimuli and prone to chronic inflammatory output (54, 55).

The NLRP3 inflammasome is also upregulated with age, primed by NF-κB and activated by mitochondrial ROS, potassium efflux, and lysosomal destabilization, leading to caspase-1-dependent maturation, IL-1β and IL-18 secretion and pyroptotic cell death (pro-inflammatory programmed cell death) (50, 56–58). These processes are further compounded by impaired mitophagy, resulting in dysfunctional mitochondria accumulation and sustained inflammasome activation (54, 58).

Macrophage polarization is influenced by the JAK-STAT3 axis, which regulates pro-inflammatory (M1) and anti-inflammatory (M2) phenotype balance (59, 60). In aging, dysregulated JAK-STAT3 signaling shifts phenotype and pro- and anti-inflammatory outputs, contributing to tissue fibrosis and impaired inflammation resolution (49, 61). Another aged macrophage hallmark is reduced engulfing and clearance of apoptotic cells (efferocytosis) and tissue repair, leading to the accumulation of such apoptotic cells and DAMPs increasing chronic inflammation (50, 62). Aging macrophages respond to and propagate SASP, creating a feed-forward inflammation loop (49, 63) resulting in transition from acute, self-limited inflammation to persistent state, characteristic of inflammaging.

### 3.2 Neutrophils: altered function and NETosis

Neutrophils also undergo profound changes with age with altered trafficking, impaired phagocytosis, and increased pro-inflammatory mediator release (64, 65). While neutrophil counts may increase with aging, their functional capacity declines, with defects in chemotaxis, phagocytosis, and microbial killing (66). Sapey et al. demonstrated impaired neutrophil chemotaxis with aging, while Wenisch et al. showed reduced phagocytic capacity (64, 66). Inflammaging is associated with an increased propensity for NETosis, the release of web-like neutrophil extracellular traps (NETs), driven by heightened inflammatory signaling and activation of enzymes such as PAD4 (12). However, senescent neutrophils exhibit reduced capacity to release NETs, instability in NET structure, and decreased DNase activity, leading to impaired pathogen clearance and increased risk of tissue damage and fibrosis (12, 67). This could create a biphasic pattern in aging where NETosis is amplified in early phase by chronic inflammation but diminished later by neutrophil senescence leading to ineffective pathogen clearance and tissue damage risk.

### 3.3 Dendritic cells: impaired tolerance and antigen presentation

Dendritic cells (DCs), essential for bridging innate and adaptive immunity through antigen presentation and cytokine production, display a pro-inflammatory phenotype during aging with increased IL-6 and TNF-α levels but reduced anti-inflammatory cytokines and type I interferons secretion (68, 69). This reduces their antigen presentation capacity, affecting T cell priming and tolerance maintenance contributing to chronic inflammation and self-tolerance breakdown. These changes are linked to epigenetic reprogramming and altered PRR signaling, further exacerbating age-associated immune dysfunction (69, 70).

### 3.4 Systemic and clinical implications

A cumulative effect of age-related immune alterations is a state in which innate immune cells remain persistently activated, continuously releasing inflammation-promoting molecules even without infection, but showing a reduced ability to respond effectively to new or acute challenges (4, 55, 68).This paradox of chronic activation coupled with varying levels of functional paralysis underlies the increased susceptibility to infections, poor vaccine responses, and heightened risk of chronic inflammatory diseases observed in older adults (4, 7, 51, 55, 68). These could modulate individual susceptibility to inflammaging and related immune diseases (51). **Table 2** provides an overview of innate immune changes and underlying molecular pathways in inflammaging.

**Table 2:** Key innate immune changes in inflammaging and molecular pathways.

Cell type	Age-related alteration	Molecular pathways involved	Clinical consequences	References
Macrophages	Increased basal cytokines, impaired polarization	NF-κB, NLRP3 JAK/STAT3	Chronic inflammation, tissue fibrosis	(4, 49–51, 54, 55, 58, 61)
Neutrophils	Impaired chemotaxis and NETosis, altered microbial killing	PAD4, ROS, cytokines	Infection risk, tissue damage, fibrosis	(12, 68)
Dendritic cells	Increased pro-inflammatory cytokines, reduced tolerance, impaired antigen presentation	PRR signaling, epigenetic changes	Loss of tolerance, chronic inflammation	(68–70)
HSCs/monocytes	Myeloid bias, inflammatory monocyte expansion	IL-1, IL-6, TNF-α, STAT3	Impaired adaptive immunity	(12, 68, 70, 166)

In summary, inflammaging fundamentally reprograms innate immunity through a convergence of mitochondrial, transcriptional, and epigenetic mechanisms. These changes render innate immune cells both hyper-inflammatory and functionally compromised, fueling chronic inflammation and immune dysfunction cycle that characterizes age-related diseases (4, 49, 50, 68).

## 4 Inflammaging impact on adaptive immunity

Aging and chronic inflammatory environment of inflammaging can remodel adaptive immune system, affecting both T and B lymphocyte compartments through changes in cellular composition, function, metabolic programming, and epigenetic regulation. These alterations reshape immune surveillance, cytokine production, tolerance and autoreactivity balance, with far-reaching consequences for immune homeostasis.

### 4.1 T cell phenotypic remodeling and functional shifts

A defining feature of adaptive immune aging is a diminished naïve T cell pool, a consequence of thymic involution and reduced thymopoiesis, leading to a reduced T cell receptor (TCR) repertoire diversity and a memory and effector phenotype shift (4, 6, 71–75). This is especially pronounced in CD8^+^ T cells, which show greater naïve cell loss and terminally differentiated, senescent, exhausted subset accumulation. Senescent T cells, particularly within the CD8 compartment, lose key components of their TCR signaling machinery but acquire natural killer (NK)-like properties, including expression of NK cell receptors and cytotoxic mediators such as granzyme B and perforin (4, 74). These cells are not merely dysfunctional but can also be activated in an antigen-independent manner by cytokines, contributing to the pro-inflammatory milieu of inflammaging.

Senescent (CD28−, KLRG1+) and exhausted T cell expansion reduces cytotoxicity and impairs pathogen/tumor clearance (76–81). This T cell exhaustion driven by inflammaging can cause increased susceptibility to infections, reduced vaccine efficacy, higher autoimmunity risk, and chronic inflammatory diseases. T cell differentiation and subset dysregulation in aging shows complex, context-dependent changes. Studies showed that aging impairs T cells’ ability to mount new Th1 responses to pathogens in older adults with reduced capacity to produce Th1 cytokines (IL-2, IFN-γ) when challenged with novel antigens or vaccines (82, 83). However, studies in older adults (mean age 85 years) show preservation or even increase in basal pro-inflammatory Th1 and Th17 cytokine levels, while anti-inflammatory Th2 cytokines (IL-4, IL-10) decline (5, 84, 85). These findings suggest that immunosenescence may reduce responses to new antigens, while inflammaging may contribute to elevated baseline inflammatory cytokines. This could explain why aging T cells appear to produce more inflammatory mediators at rest but respond poorly when challenged with new pathogens.

Aged T cell functions are further shaped by metabolic and epigenetic reprogramming. Naïve T cells rely on mitochondrial oxidative phosphorylation to maintain quiescence, but upon activation, they switch to glycolysis and upregulate nutrient uptake via mTOR pathway (79, 86). During aging, T cells exhibit impaired mitochondrial function, increased ROS production, and altered mTOR signaling, which skews differentiation toward short-lived effector phenotypes and limits memory formation (79, 86). Epigenetically, aged T cells display increased chromatin accessibility at effector loci (e.g., BATF, T-bet) and decreased accessibility at genes involved in quiescence and self-renewal, such as TCF1 and LEF1 (72, 73). These changes are reinforced by DNA methylation and histone modifications, driving quiescence loss and a bias toward effector and exhausted states.

T cell cytokine production is also altered in aging and inflammaging. While some studies report a decline in IFN-γ production by T cells in older men, others show stable or even increased IFN-γ, particularly in older women and in the context of chronic inflammation or immune disease (75, 87–89). The Th1/Th2/Th17 balance is perturbed, with a relative preservation or increase in Th17 and Th1 cytokines (IL-17, IFN-γ, TNF-α), and a reduction in anti-inflammatory cytokines such as IL-4 and IL-10 (71, 88–90). Studies show age-related increases in pro-inflammatory serum IL-6 levels from approximately 1.5 pg/mL in young adults (<30 years) to 2.0 pg/mL in middle-aged (30–60 years) to 3.5 pg/mL in older adults (>60 years), with strong positive correlation (r=0.664, p<0.001) (91). Similarly, TNF-α rises from approximately 1.5 pg/mL in young to 1.8 pg/mL in middle-aged to 1.9 pg/mL in older adults (r=0.368, p=0.003), and IL-1β increases from 0.5 pg/mL to 2.0 pg/mL to 2.3 pg/mL across the same age groups (r=0.281, p=0.027) (91). Notably, IL-17 shows an opposite trend, decreasing significantly with age from approximately 11 pg/mL in young adults to 3 pg/mL in middle-aged to 2 pg/mL in older adults (r=-0.427, p=0.005), while IL-23 remains relatively stable across age groups (91). Anti-inflammatory IL-10 increases with age, though the magnitude is modest (91). The Th17/Treg balance could show age-dependent changes: basal CD4+IL23R+ (Th17) cells continuously increase with age, while the Th17/Treg ratio increases at rest in older individuals but paradoxically decreases after stimulation, accompanied by elevated Foxp3 mRNA and IL-10 protein expression (92).

This shift with age promotes chronic inflammation and tissue damage. Regulatory T cells (Tregs) increase in frequency with age but often display impaired suppressive function and altered cytokine profiles, sometimes acquiring pro-inflammatory characteristics (71, 73, 74, 88).

T cell exhaustion, characterized by sustained expression of inhibitory receptors (PD-1, CTLA-4, KLRG1) and loss of effector function, is a hallmark of chronic antigenic and inflammatory stimulation in aging (4, 74, 90, 93). Exhausted T cells accumulate in tissues and peripheral blood contributing to impaired pathogen and tumor clearance, but also to chronic inflammatory state maintenance. The central exhaustion program regulator TOX transcription factor enforces epigenetic and transcriptional changes locking T cells in this state (74, 93).

### 4.2 B cell subset dynamics and autoreactivity

B cell development and function are also markedly altered with age. There is a decline in early B cell progenitors, impaired lineage commitment, and reduced output of naïve B cells from bone marrow, driven by intrinsic epigenetic changes and increased protein turnover of key transcription factors such as E2A (E47) (75, 94, 95). Mature B cells show reduced turnover, impaired class-switch recombination, somatic hypermutation, diminished antibody diversity and affinity (94–96). These defects are compounded by altered BCR signaling, increased metabolic activity (glucose uptake, OXPHOS, FAO), and persistent antigenic stimulation.

A key feature of B cell aging is age-associated B cell (ABCs) expansion, a subset characterized by T-bet and CD11c expression, pro-inflammatory cytokine production (IL-6, TNF-α), and propensity for autoreactivity (75, 89, 94, 96). Age-associated B cells (ABCs, DN B cells) and other memory B cell subset expansions can polarize T helper cells toward inflammatory phenotypes (Th1, Th17) via antigen presentation, autoantibodies and pro-inflammatory cytokine secretion, amplifying inflammatory circuit of inflammaging (75, 89, 94, 97, 98). B regulatory cells (Bregs), which normally suppress inflammation through IL-10, IL-35, and TGF-β, may be reduced or functionally impaired in the older adults, further favoring inflammation (94). B subsets and cytokine environment alterations can reduce high-affinity, protective antibody generation and impair memory B cell and plasma cell function (97, 98).

Autoantibody production increases with age, reflecting both a breakdown in central and peripheral tolerance and pro-inflammatory environment of inflammaging (71, 96, 99). Elevated TNF-α from aged B cells is negatively correlated with activation-induced cytidine deaminase (AID) expression, hindering class-switch recombination and somatic hypermutation, but promoting autoreactive clone survival and expansion (96, 99). The IgG autoantibodies from older individuals often display altered glycosylation and sialylation, which can modulate their pro- or anti-inflammatory effects via Fc receptor binding (99). This accumulation of autoantibodies and immune complexes, B cell subsets and B cell-derived cytokines could result in inflammatory feedback amplifying local, systemic inflammation as well as inflammaging contributing to age-related tissue damage and autoimmunity (71, 96, 99, 100).

### 4.3 T and B cell crosstalk and immune network remodeling

The interplay between B cells and T cells is increasingly recognized as a driver of adaptive immune aging. B cells can promote T cell immunosenescence through direct cell-cell contact, cytokine secretion such as IFN-γ, TNF-α, and TGF-β, and antigen presentation that skews T cell differentiation toward pathogenic effector and exhausted subsets (75, 89, 94). Conversely, T cell-derived cytokines influence B cell activation, differentiation, and antibody production, establishing a feed-forward loop that sustains chronic inflammation and immune dysfunction (75, 89, 94).

Collective T and B cell alterations with aging suggest inflammaging can be characterized by shifts toward pro-inflammatory phenotypes, sustained IFN-γ and Th17 cytokines, exhausted and senescent lymphocyte accumulation, and increased autoreactive antibody production, underpinned by metabolic and epigenetic remodeling. **Table 3** provides an overview of age-associated changes in adaptive immune cells, and **Table 4** summarizes T and B cell cytokine production changes with inflammaging.

**Table 3:** Age-associated changes in adaptive immune cells and molecular pathways.

Cell type/subset	Age-related alterations	Molecular pathways & mechanisms	References
Naïve CD4^+^ T cells	Reduced pool size, impaired homeostasis, increased IL-7-driven AKT activation	Decreased FOXO1 activity reduces IL-7R/CCR7; reduced TCR signaling due to miR-181a loss and increased DUSP6; sustained AKT-mTORC1 and ERK signaling skews toward short-lived effector differentiation; increased chromatin accessibility at effector gene loci (BATF, T-BET); telomerase activity loss and telomere shortening;	(4, 6, 72–75, 79, 86)
Naïve CD8^+^ T cells	Reduced quiescence, increased homeostatic proliferation, effector-like features	Reduced NRF1 expression; decreased IL-7R/CCR7 expression due to FOXO1 inhibition; excessive AKT activation; epigenetic remodeling (DNA methylation, histone modification) leads to central memory-like phenotype	(4, 6, 72–75, 79, 86)
Memory CD4^+^ T cells	Effector memory cells expansion, increased IFN-γ production, proliferative capacity reduced, telomere erosion	Increased accessibility to BATF and T-BET, EOMES transcription factors; demethylation and overexpression of effector genes; increased IL-15 sensitivity; altered metabolic programming (reduced glycolysis, increased ROS)	(4, 6, 72–75, 79, 86–89)
Memory CD8^+^ T cells	Senescent/exhausted phenotype accumulation, inhibitory receptor upregulation (PD-1, CTLA-4, KLRG1), reduced cytotoxicity	TOX-driven exhaustion program; persistent antigenic stimulation; increased SASP; mTORC1 hyperactivation	(4, 6, 72–75, 79, 86–89, 93)
Effector CD4^+^ T cells	Skewed differentiation toward Th17/Th1, increased IL-2Rα, TIM3, granzyme B expression	Sustained AKT-mTORC1 and ERK signaling; BLIMP1, RUNX3 upregulation; downregulation of TCF1, LEF1, IL-7R, CD62L, CD27; increased miR-21 expression; impaired lysosomal activity; increased SIRT1 expression leads to replication stress and cell cycle arrest	(4, 6, 72–75, 79, 86–89)
Regulatory T cells (Treg)	Increased natural Treg frequency, decreased inducibility from precursors, impaired suppressive function, altered cytokine profile	Increased Foxp3 expression; altered IL-10 and TGF-β signaling; epigenetic changes affecting Treg stability	(6, 71, 73, 74, 88, 94)
B cell progenitors	Reduced early B cell progenitors (EBPs), impaired lineage commitment	Epigenetic changes in HSCs and B cell precursors; increased ERK MAPK activity; reduced RAGs and surrogate light chain (lambda-5) expression; impaired Notch signaling	(75, 94, 95)
Mature B cells	Reduced turnover, impaired class-switch recombination and somatic hypermutation, diminished antibody diversity/affinity	Reduced E2A due to increased mRNA degradation; altered BCR signaling; increased Glut1 expression and glucose uptake; persistent antigenic stimulation leads to age-associated B cell expansion (ABCs); epigenetic changes affecting memory/plasma cell differentiation	(75, 94–96)
Plasma cells	Reduced long-lived plasma cells, decreased antibody secretion	Impaired metabolic reprogramming; decreased transcription factors expression (e.g., BLIMP1, XBP1); reduced survival signals (BAFF, APRIL)	(75, 94–96)
Memory B cells	Reduced pool size, impaired recall responses, increased autoreactivity, expansion of ABCs	Epigenetic remodeling; altered BCR and TLR signaling; upregulation of pro-inflammatory cytokines (TNF-α, IL-6); impaired class-switch recombination	(75, 89, 94–96)
Age-associated B cells (ABCs)	Expansion in aged individuals, pro-inflammatory/autoimmune phenotype, increased cytokine and autoantibody production	TLR7/9 signaling and persistent antigenic stimulation; metabolic reprogramming (increased glycolysis, OXPHOS); epigenetic changes reinforce inflammatory gene expression; impaired negative selection of autoreactive clones	(75, 89, 94–96)

**Table 4:** Cytokine production by T and B cell subsets in inflammaging.

Subset	Hallmark cytokines produced	Role in inflammaging and aging-related shift	Plasticity/Notes	References
Th1	IFN-γ, TNF-α, IL-2	IFN-γ and TNF-α maintained or increased with age, especially women and with chronic inflammation; can cause chronic inflammation, tissue damage	Th1/Th17 hybrid cells in chronic inflammation; IFN-γ by CD8^+^ T cells and ABCs	(75, 87–90)
Th2	IL-4, IL-5, IL-13	Reduced differentiation and cytokine output; IL-4 and IL-13 may cause chronic inflammation	Th2/Th17 hybrid states in chronic inflammation	(75, 88–90)
Th17	IL-17A, IL-17F, IL-21, IL-22, TNF-α, IL-6	Relative preservation or increase with age; Th17 dominance and increased IL-17/IL-22, tissue damage, autoimmunity	Highly plastic; can convert to Th1 or Th2; Th17/Th1 and Th17/Th2 hybrid cells	(75, 88–90)
Treg	IL-10, TGF-β, IL-35	Increased frequency in aging with impaired suppressive function and altered cytokine profile	Can lose FoxP3 and acquire effector cytokine production	(71, 73, 74, 88)
Tfh	IL-21, IL-4, IL-10	Supports B cell help and antibody production; may be reduced with age	Shares features and cytokines with Th17; promote autoimmunity	(75, 89, 94)
Cytotoxic CD8^+^ T cells	IFN-γ, TNF-α, perforin, granzyme B	Accumulate as senescent/exhausted cells with altered cytokine output	Senescent CD8^+^ T cells can gain NK-like properties	(4, 74, 75, 87, 88)
Memory B cells (Be1, Be2, GM-CSF^+^, IRA B cells)	IL-6, TNF-α, IFN-γ, GM-CSF, LT, IL-10	Memory B cells and ABCs expand with age, producing more pro-inflammatory cytokines can promote inflammaging and autoimmunity	B cell cytokine profile depends on activation context; Be1 (promote Th1), Be2 (promote Th2); IRA B cells (GM-CSF^+^) support innate responses	(75, 89, 94, 96)
Plasma cells	IL-10, (occasionally IL-6, TNF-α)	Reduced IL-10 in aged plasma cells; impaired anti-inflammatory feedback	Survival and cytokine production depend on signals from Tfh	(75, 89, 94, 96)
Age-associated B cells (ABCs)	IL-6, TNF-α, IFN-γ, GM-CSF, autoantibodies	Expanded in aging and chronic inflammation; pro-inflammatory cytokines and autoantibodies; can promote inflammaging and autoimmunity	Driven by TLR7/9 and persistent antigenic stimulation; T-bet^+^, CD11c^+^ phenotype	(75, 89, 94, 96)

## 5 Gender differences, estrogen and inflammaging

Women exhibit stronger adaptive immune responses and higher autoimmune prevalence than do men, while men show greater monocyte activation and systemic inflammaging (101, 102). Immune cells express estrogen and androgen receptors, through which sex hormones modulate TLR signaling, type I IFN responses, and cytokine production, contributing to observed gender differences (101).

Human studies demonstrate sex differences in immune aging, though findings are not universally consistent. While studies suggest men generally exhibit a more pro-inflammatory “inflammaged” profile with aging and women maintain stronger adaptive immunity into later life, this narrative is not without contradictions.

Studies profiled blood immune cells from individuals 22–93 years old and found that after age 65, men exhibited higher innate and pro-inflammatory activity and lower adaptive (T- and B-cell) activity than women (103).Immune aging changes accelerated earlier in men, beginning at ages 62-64, while comparable changes in women occur from 66–71 years onward (93).

Similarly, Huang et al. showed that men aged 60–80 had higher inflammatory monocytes, increased innate signaling, and steeper naïve T cell decline compared to women, who retained greater adaptive immune function into older age (104). Studies noted that females generally display stronger and longer-lasting adaptive immune responses across adulthood and into old age (105). These women also experienced gradual and slower immune decline, while males demonstrated earlier and more marked reduction in T- and B-cell immunity and increased inflammation-driven disease risk. Giefing-Kröll et al. highlighted that men exhibit stronger and earlier decrease in adaptive immune cells with increased innate activity as they age, whereas women maintain adaptive responses longer, with greater immune decline occurring after menopause (106). However, it is important to note that the heterogeneity in findings could reflect differences in study populations, measurement techniques, and environmental factors.

Aged women, especially post-menopause, show rising inflammation with increased C-reactive protein, TNF-α, IL-6, ceruloplasmin, complement C3, fibrinogen, and β2-microglobulin, though this occurs later than in men (107, 108). In animal studies, similar sexual dimorphism was observed. Females tend to mount stronger innate and adaptive responses than males and this difference is generally observed regardless of age. This can become more pronounced after sexual maturity and with aging as shown in female mice generating higher antibody responses to pathogens, while male mice accumulate more inflammatory monocytes with age (109, 110).

Men’s immune cells decline 5–6 years earlier than women’s (111). These differences could stem from hormonal and genetic factors as women have two X chromosomes enriched for immune genes and estrogen, whereas men have higher levels of androgens. Bernardi et al. study (112) reported in healthy adults aged 22–45 years, men had median cytokine levels of IL-1β around 45 pg/ml versus 5 pg/mL in women, IL-6 around 42 pg/mL versus 15 pg/mL in women, and TNF-α about 90 pg/mL in men versus 25 pg/mL in women. However, these data showed extreme variability with ranges extending to very high levels (>500 pg/mL for IL-1β, >300 pg/mL for IL-6), suggesting potential subclinical or lifestyle conditions that may contribute more than sex differences alone.

Men over 65 years exhibited higher IL-6 levels and related markers than age-matched women, correlating with reduced longevity (113). In this study, men aged 51–60 years had IL-6 levels of 0.95 mg/dL versus 0.88 mg/dL in women, and TNF-α of 2.50 mg/dL versus 2.45 mg/dL. In the 61-70-year group, IL-6 levels were 0.88 mg/dL in men and 0.92 mg/dL in women, while TNF-α levels were 2.45 mg/dL and 2.40 mg/dL, respectively.

The most robust circulating biomarkers of inflammaging such as IL-1β, IL-6, TNF-α, and CRP are consistently associated with age-related disease risk with levels modulated by both sex and hormonal status (4, 101, 114). Studies demonstrate that healthy adult men generally have higher circulating IL-1β, IL-6, and TNF-α than women, a difference only partially explained by sex hormones (4, 101, 114).

Females have longer telomeres and higher telomerase activity than males, a difference established at birth and maintained throughout life, largely due to estrogen’s protective effects on telomere maintenance and protection from oxidative stress (101, 115). Leukocyte telomere length, an established biomarker of inflammaging, reflects this sexual dimorphism and may contribute to the delayed onset of age-related diseases observed in women compared to men (115).

Overall data show males accumulate more age-related inflammatory changes (innate cells, cytokines) with decreased adaptive function earlier, whereas pre-menopausal women maintain robust adaptive immunity (T/B cell responses) that can delay inflammaging (103, 110). These gender**/**sex differences are shaped not only by hormones (i.e. estrogen) but also by genetic and epigenetic factors, including X-linked immune gene mosaicism, microRNA expression and telomerase activity (101, 103, 104, 110). Environmental and lifestyle factors, such as exposure to pollutants, diet, and stress, can further accelerate inflammaging in a sex-dependent manner, with men generally more susceptible to pollutant-induced inflammation and women more affected by indoor pollutants and endocrine disruptors (116).

### 5.1 Estrogen and cellular senescence in the context of inflammaging

Cellular senescence plays a pivotal role in the development of inflammaging by contributing to the accumulation of senescent cells and pro-inflammatory SASP secretion. Many senescence inducers act through DNA damage and DNA damage response (DDR) pathways, investigating sex differences in these molecular mechanisms has revealed important distinctions that may underlie differential inflammaging patterns between males and females.

Several studies demonstrated estrogen can exert protective effects against genotoxic stress and senescence-related pathways. Estrogen reduces oxidative stress by suppressing mitochondrial ROS release through both direct mitochondrial interaction and activation of mitochondrial estrogen receptors, as shown in skeletal muscle cells, primary neurons, and cerebral endothelial cells (117–119) Estrogen can prevent DNA fragmentation by hydrogen peroxide and etoposide in mouse skeletal muscle cells (120) and promote telomerase expression and activity in human leukocytes, vascular smooth muscle cells, endothelial progenitor cells, as well as in postmenopausal arterial tissue, supporting telomere maintenance to delay cellular senescence (121, 122).

At the level of checkpoint control, estrogen has been shown to inhibit key DDR regulators. In human ER-positive breast cancer cells, estrogen suppresses Chk1, γH2AX, and ATR expression and activity, which downregulates p21, a central senescence effector (123). Inhibition of ER-α in both normal and malignant mammary epithelial cells leads to increased SA-β-gal staining and dephosphorylated RB family proteins, indicating senescence induction (124). Estrogen can upregulate WRN expression in human breast cells, a DNA helicase involved in genome stability and senescence prevention (125).

The absence of estrogen, as seen in postmenopausal women or ovariectomized (OVX) animals, is associated with accelerated senescence and heightened age-related inflammation. In postmenopausal individuals, blood-derived macrophages exhibit reduced markers of anti-inflammatory (M2) activity (126). In OVX mice, bone marrow mesenchymal stem cells display increased p53 and p21 expression, SASP secretion, JAK/STAT pathway activation and decreased IFN-γ expression from splenic lymphocytes (127, 128). Combined administration of IL-7 and IL-15 in OVX female mice induces antigen-independent production of IL-17A and TNF-α by bone marrow memory T cells, reflecting possible chronic low-grade inflammation state (129). These estrogen-deficiency–driven immune alterations not only accelerate tissue aging but also contribute to the persistent inflammatory milieu characteristic of inflammaging.

Estrogen replacement can reverse several of these deleterious effects. In postmenopausal women and OVX mice, estrogen administration downregulates p53 and p21 expression, SASP output, and JAK/STAT signaling (127). Estrogen also reduces SASP markers including GDF-15, IFN-γ, MCP-1, TNF-α, and matrix metalloproteinases (MMP-2, MMP-9) in both whole blood and isolated platelets (130, 131).

However, estrogen can activate signaling cascades that intersect with SASP-promoting pathways, including p38 MAPK, PI3K, and mTOR, particularly in aged tissues (132). This complexity underscores the dualistic nature of estrogen signaling while estrogen generally delays cellular senescence onset and suppresses SASP under physiological conditions, it may promote pro-senescent or inflammatory outcomes under specific pathological conditions.

These findings establish that estrogen exerts context-dependent effects on cellular senescence and inflammaging, providing protection during premenopausal years through contributions to genomic stability, mitochondrial function, and inflammatory control, but transitioning to accelerated senescence and increased inflammatory burden following estrogen deficiency at menopause.

### 5.2 Inflammaging: is there a difference between young and menopausal women vs men

Multiple studies report postmenopausal women have higher IL-6 levels, an inflammaging marker, and other pro-inflammatory markers than younger premenopausal peers. Healthy nonobese women aged 22–63 years showed increasing serum IL-6 with age, with postmenopausal women having IL-6 levels of 5.2 pg/mL versus 2.6 pg/mL in premenopausal women, higher TNF-α (8.1 vs. 6.7 pg/mL), and slightly lower IL-1β (0.73 vs. 0.81 pg/mL) (133). Notably, estrogen deprivation after menopause enhanced IL-6 levels from PBMC. Higher IL-6 has been reported in postmenopausal women (4.4 pg/mL) compared to reproductive-age women (2.1 pg/mL), while TNF-α decreased after menopause (2.1 pg/mL postmenopausal vs. 3.4 pg/mL reproductive) (134).

Comparing women to men across age groups reveals additional complexity. In women aged 51–60 years (10 of 11 were menopausal), IL-6 averaged 0.88 mg/dL compared to 0.95 mg/dL in men, while in the 61–70 years group, women had 0.92 mg/dL and men 0.88 mg/dL (114). For TNF-α in the 51–60 age range, women had 2.45 mg/dL and men 2.50 mg/dL; in 61–70 years, women had 2.40 mg/dL and men 2.45 mg/dL (114). Sex-related differences in pro-inflammatory cytokine levels are most evident for IL-6 in the 51-60-year age range, where men exhibit notably higher concentrations than women, but the gap narrows and reverses by 61–70 years (114).

Young women in their 20s-40s demonstrate more robust adaptive immunity and lower baseline inflammatory markers compared to age-matched men. Men exhibit significantly higher levels of IL-6 (42 vs. 15 pg/mL), IL-1β (45 vs. 5 pg/mL), and TNF-α (90 vs. 25 pg/mL) than women (112). IL-6 increases with age primarily in men, conferring a longevity disadvantage (135). Both IL-6 and CRP, key biomarkers of inflammaging, remain lower in healthy premenopausal women than age-matched men, possibly due to estrogen’s modulation of cytokine production. Young women also show stronger immune reactivity, including higher antibody titers and more vigorous T-cell responses to pathogens and vaccines than men (105, 109, 136).

Thus, menopause could induce a pro-inflammatory transition, as estrogen loss leads to higher IL-6 and TNF-α output, partly explaining increased chronic disease incidence after menopause. Young women clear infections more efficiently with greater susceptibility to autoimmune and inflammatory conditions, while men’s higher baseline inflammatory cytokine levels established early in adulthood could set the stage for accelerated inflammatory aging compared to premenopausal women.

### 5.3 Environmental and lifestyle influences on inflammaging by sex differences

Environmental and lifestyle factors interact with sex to shape inflammaging, though additional data are still needed. Unhealthy lifestyle (obesity, poor diet, smoking, chronic stress) amplifies inflammaging in both sexes, but some effects are sex-specific. Obesity represents a major inflammatory source via fat tissue production of IL-6 and TNF-α. Diet-induced obesity models show that male rodents develop higher systemic inflammation than females (137). Obese males have greater pro-inflammatory responses such as higher M1 macrophages in adipose tissue and 2–3 fold higher TNF-α and IL-6 levels than females on high fat diet, while obese females expressed more anti-inflammatory M2 macrophages (138).

Stress and sleep patterns also differ by sex and affect immunity. Chronic psychosocial stress may elevate pro-inflammatory cytokines more in men, though findings are mixed (139). Environmental toxins and pollutants also have sex-differential effects. Men historically have higher exposures to industrial chemicals and smoking, while women have greater exposure to household/outdoor pollutants and endocrine disruptors, which can have an impact on inflammatory responses (140). Diet composition and microbiome interact with sex hormones to alter immune responses (141). Mediterranean-type diets may benefit both sexes, but men tend to accumulate more visceral fat leading to inflammatory environment than women for the same diet/caloric load (142). Men with risk factors (obesity, sedentary life, smoking) often show higher IL-6/CRP levels than comparably affected women (143), while higher CRP concentrations in premenopausal women appear to result from their greater accumulation of subcutaneous fat compared to men (144). Women, by contrast, may manifest environmental stress as different disease risks (e.g. autoimmunity, bone loss) but without as high basal inflammation (145). In summary, lifestyle factors can influence inflammaging differently by sex, though both sexes can suffer inflammaging from poor lifestyle and external factors.

## 6 Inflammaging in female autoimmunity

Female-predominant autoimmune diseases, including systemic lupus erythematosus, multiple sclerosis, rheumatoid arthritis, and Hashimoto’s thyroiditis, arise from complex interactions of genetic, epigenetic, hormonal, and environmental factors (109, 146).

Inflammaging is now recognized as a critical background process that amplifies susceptibility to and progression of autoimmunity in women (101, 109, 136). Unlike the episodic chronic inflammation seen in active autoimmune disease, inflammaging involves elevated circulating pro-inflammatory cytokines (IL-6, IL-1β, TNF-α, IFN-γ, CRP), accumulation of senescent immune cells with SASP, altered redox and metabolic states (12, 145).

At the molecular level, inflammaging in female autoimmunity involves persistent NF-κB and JAK-STAT pathway activation, chronic stimulation of PARs such as TLR7, which is X-linked and more expressed in females, expansion of late-differentiated effector T cells and age-associated B cells (ABCs) (12, 147, 148).The ABCs that are more prevalent in aging women secrete high er IL-6, TNF-α, and autoantibody levels, perpetuating SASP and amplifying tissue damage (101, 147). These findings may be suggestive for the overlap between inflammaging and autoimmunity (12), and understanding inflammaging, sex hormones, and autoimmunity intersection is key for intervening therapies.

## 7 Inflammaging mirroring out: skin implications

The skin provides a unique window into inflammaging processes, serving as both sentinel and contributor to systemic inflammation. Sexual dimorphism extends to skin immunity, with androgens and estrogens differentially affecting skin immune responses (149). Inflammaging in skin is driven by senescent cell accumulation, chronic pro-inflammatory mediator production, extracellular matrix disruption, and skin-resident immune system dysregulation. These processes are amplified by age-related skin microbiome changes that contribute to skin aging phenotypes, impaired barrier function, and increased disease susceptibility.

Senescent dermal fibroblasts accumulate with age and after repeated exposure to UV radiation and oxidative damage, secreting SASP rich in pro-inflammatory cytokines including IL-6, IL-8, TNF-α, and matrix metalloproteinases (MMPs). These SASP factors amplify local inflammation, degrade collagen, and disrupt extracellular matrix (ECM) homeostasis, resulting in dermal thinning, elasticity loss, wrinkles and impaired wound healing (150–152). SASP activates JNK and AP-1 signaling, further upregulating MMPs and accelerating collagen breakdown (150, 151). Chronic inflammation with persistent SASP signaling creates a microenvironment conducive to carcinogenesis and other age-related skin pathologies (151, 152).

Keratinocytes also undergo senescence with age and repeated UV exposure secreting pro-inflammatory cytokines and inducing fibroblast senescence via IL-1 signaling. This establishes paracrine feedback loop that perpetuates tissue inflammation and ECM degradation (150–152). Loss of IGF-1 expression in senescent fibroblasts further impairs keratinocyte survival and differentiation, exacerbating epidermal atrophy and barrier dysfunction (153). Inflammaging can impact on collagen dynamics, pigmentation changes and further impairing epidermal renewal. Senescent fibroblasts display reduced collagen gene expression (COL1A1, COL3A1, COL4A1) and increased MMPs secretion, leading to decreased collagen deposition, increased collagen fragmentation, and weakened ECM (150, 154). This not only results in signs of aging but also impairs wound healing and structural resilience (155, 156).

Skin-resident immune cells are reshaped by inflammaging. Langerhans cells, specialized tissue-resident macrophages with dendritic cell-like antigen-presenting functions, decline in numbers and function with age, impairing immune surveillance and contributing to increased susceptibility to infections and inflammatory dermatoses (150, 153). Skin-resident T cells show age-related compartmental heterogeneity, with the Koguchi-Yoshioka et al. study demonstrating increased CD8+ T cell infiltration and reduced CD4 to CD8 ratio in aged epidermis (157), while Zuelgaray et al. found increased CD4 to CD8 ratio in whole skin explant cultures containing both epidermis and dermis (158), suggesting different skin layers could exhibit distinct T cell distributions that collectively contribute to local inflammation with cytokines entering systemic circulation to promote systemic inflammaging (150, 156).

Inflammaging and skin microbiome dysregulation can interact bidirectionally. With age, protective commensal species in microbiome such as Cutibacterium decline, while pro-inflammatory taxa such as Corynebacterium and Streptococcus expand (159, 160). This dysbiosis alters skin surface pH, lipid composition, and barrier function, creating permissive environment for chronic, low-grade inflammation accelerating collagen degradation and impairing wound healing (159, 160). Age-related microbiome shifts potentially undermine regulatory T cell and antimicrobial peptide protective mechanisms (156, 161).

In addition to these intrinsic factors, environmental and lifestyle factors including UV exposure, pollution, poor sleep and diet accelerate skin inflammaging by promoting oxidative stress, DNA damage, skin barrier and microbiome disruption (150). The cumulative impact is premature skin aging characterized by wrinkles, dryness, impaired barrier function, and a heightened risk of irritation and neoplasia.

## 8 Conclusion

Our understanding of inflammaging mechanism and responses is rapidly developing. This chronic, low-grade, systemic inflammatory state links aging to immune dysfunction, tissue degeneration, and increased disease susceptibility. Inflammaging is mechanistically distinct from but intimately connected to immunosenescence, driven by intrinsic cellular senescence and extrinsic environmental factors. The interplay between innate and adaptive immune alterations, sex hormones particularly estrogen, and genetic and epigenetic regulators shapes inflammaging’s trajectory and tissue-specific manifestations including skin aging. Cellular senescence, SASP production, immune cell reprogramming, and microbiome shifts converge to amplify these processes, creating systemic repercussions that extend beyond individual organs. Thus, even in our early understanding of inflammaging’s complexity, the pathways that drive it remain intriguing targets for therapeutic intervention.
